# MSCs Become Collagen-Type I Producing Cells with Different Phenotype in Allogeneic and Syngeneic Bone Marrow Transplantation

**DOI:** 10.3390/ijms22094895

**Published:** 2021-05-05

**Authors:** Robert Maximilian Rusch, Yoko Ogawa, Shinri Sato, Satoru Morikawa, Emi Inagaki, Eisuke Shimizu, Kazuo Tsubota, Shigeto Shimmura

**Affiliations:** 1 Department of Ophthalmology, Keio University School of Medicine 35 Shinanomachi, Shinjuku, Tokyo 160-8582, Japan; robert.rusch@keio.jp (R.M.R.); shinri.sato259@gmail.com (S.S.); emiharada512@yahoo.co.jp (E.I.); ophthalmolog1st.acek39@keio.jp (E.S.); tsubota@z3.keio.jp (K.T.); 2 Department of Dentistry and Oral Surgery, Keio University School of Medicine 35 Shinanomachi, Shinjuku, Tokyo 160-8582, Japan; morikawa@keio.jp

**Keywords:** mesenchymal stem cells (MSCs), graft-versus-host disease (GVHD), lacrimal gland

## Abstract

Mesenchymal stem cells (MSCs) have been widely used in therapeutic applications for many decades. However, more and more evidence suggests that factors such as the site of origin and pre-implantation treatment have a crucial impact on the result. This study investigates the role of freshly isolated MSCs in the lacrimal gland after allogeneic transplantation. For this purpose, MSCs from transgenic GFP mice were isolated and transplanted into allogeneic and syngeneic recipients. While the syngeneic MSCs maintained a spherical shape, allogeneic MSCs engrafted into the tissue as spindle-shaped cells in the interstitial stroma. Furthermore, the MSCs produced collagen type I in more than 85% to 95% of the detected GFP^+^ MSCs in the recipients of both models, supposedly contributing to pathogenic fibrosis in allogeneic recipients compared to syngeneic models. These findings indicate that allogeneic MSCs act completely differently from syngeneic MSCs, highlighting the importance of understanding the exact mechanisms behind MSCs.

## 1. Introduction

Mesenchymal stem cells (MSCs) have been studied for more than five decades [1,2]. Their versatility, broad clinical potential, as well as their potential for tissue engineering results in an ongoing interest. Within a year after their first isolation and in vitro expansion in 1992, human bone marrow cells were used to treat patients [3,4]. This highlights their great diversity. MSCs have shown an incredible potential to differentiate into various types of cells. Several studies demonstrated that adult human MSCs are able to differentiate into the osteo-, chondro-, adipo-, myo-, and teno-lineage [5,6,7]. They are also very prevalent in tissue engineering also due to their potential to produce collagen [8,9]. For instance, MSCs are shown to express collagen-type I and collagen-type III when aligned with silk fibers [10,11]. They are major players in tissue repair as they are attracted to areas of inflammation or damaged vessels and act in a milieu-specific manner [12]. Furthermore, human studies showed their involvement in cartilage repair [13]. Through the promotion of angiogenesis, regeneration, and modulation of the immune system they closely mediate tissue repair [12]. The involvement of MSCs in various tissue repair is further emphasized by Stolzing et al. who showed that there might be a connection between age-related MSC decrease and frailty [14].

MSCs are commonly acquired from bone marrow and adipose tissue. For human studies, the umbilical cord tissue and placenta are also quite common [15,16]. However, several studies suggest that the MSCs’ exact phenotype is dependent on the tissue they were attained from [17,18,19,20].

While it is common to expand MSCs in vitro after isolation, several studies have shown that this changes their phenotype and function. Seemingly minor influences, such as the stiffness of the culture dish, have shown to influence their character [21]. Furthermore, Selich et al. demonstrated that out of a large number of MSCs, only a few individual cell strains survive, resulting in a de-complexed culture [22]. Consistently, cultured MSCs are used to treat steroid therapy-refractory Graft-Versus-Host Disease (GVHD) or can be used as a prophylactic to prevent GVHD [23,24,25]. In contrast, Ogawa et al. showed that freshly isolated donor MSCs are a key player in developing said disease. The interaction between mismatched MSCs and T cells induces IL-6 production. This in turn leads to pathological changes through the recruitment of IL-17 helper T cells and inflammation [26]. Further studies suggested that even cultured MSCs transplanted with hematopoietic stem cells (HSCs) that result in a matched model can result in GVHD [24,27]. Taken together, MSCs have a Janus-faced character depending on many factors. Hence, it is important to differentiate not only between the MSCs’ origin but also if they were cultured before usage.

When transplanting cells, it is important to distinguish the major histocompatibility complex (MHC) and the minor antigen matching of donor and recipient. In general, there is the syngeneic (MHC matched, minor antigen matched), allogeneic (MHC and/or minor antigen mismatched), and autologous (transplantation of the patients’ own cells) transplantation. Differences in the minor histocompatibility antigens (miHAg) underlie the GVHD development, despite MHC matching [28,29]. When these peptides are presented by MHC class I or MHC class II antigens they cause an immune reaction if the recipient does not have the same variant. Even a single nucleotide polymorphism (SNP) or deletion is sufficient to cause a reaction of cytotoxic T lymphocytes resulting in GVHD.

GVHD is a major cause of morbidity and mortality after allogeneic stem cell transplantation [30]. It is divided into an acute (aGVHD) and chronic (cGVHD) phases. GVHD is a systemic disease presenting tissue injury and inflammation through pro-inflammatory cytokine release. One of its hallmarks being skin pathology varying from lichen planus–like lesions to full sclerosis [31]. Pathogenic fibrosis is found in most target organs including the liver, intestines, and salivary and lacrimal glands [32,33,34,35].

The lacrimal gland is an exocrine tissue which is mostly responsible for secreting the tear film of the eye. It secretes the fluid through lacrimal ducts to the ocular surface. Dysfunction of the lacrimal gland due to chronic inflammation and pathogenic fibrosis results in the loss of aqueous and other products required for the ocular surface maintenance and health resulting in dry eye disease and the potential for significant surface pathology. The lack of tear fluid leads to another common pathological feature in chronic GVHD: dry eye disease [32].

The exact function of MSCs in GVHD is still relatively unexplored. Hence, the aim of this study was to investigate the role of freshly isolated MSCs in a minor antigen mismatched GVHD model. Bone marrow transplantation (BMT) was conducted to create a murine model of cGVHD. Since collagen plays an important role in GVHD and MSCs can produce collagen under certain conditions, we hypothesized that freshly isolated donor-derived MSCs are involved in cGVHD collagen production.

The MSCs were isolated from the bone marrow by FACS with a classic [26] and new approach [36,37]. The bone marrow was analyzed to see if the donor cells are homing, and the blood was analyzed to see if MSCs are circulating. The main focus, however, lies on the lacrimal gland, one of the most frequently affected organs in chronic GVHD patients. Our results suggest that MSCs navigate to the lacrimal gland and become collagen-producing cells. Thus, they might be a major contributor to fibrosis in GVHD.

## 2. Results

This study aimed to investigate the impact of donor MSCs in GVHD, focusing on the ocular area, the lacrimal gland in particular. For this purpose, MSCs from transgenic GFP expressing mice were isolated by Fluorescence-Activated Cell Sorting (FACS). This study used two approaches to achieve this. Firstly, MSCs were defined as CD45 TER-119 Sca-1^+^ PDGFRα^+^ cells [38]. This classical method removes the hemopoietic cells (CD45), erythroid cells (TER-119), and includes the stem cell antigen-1 (Sca-1) and myeloid/lymphoid cells (PDGFRα). The method from Morikawa and colleagues became well-established. The other definition also included CD45 TER-119. However, instead of Sca-1 and PDGFRα, CD73, found as an MSC marker in 2017, was included and cells positive for the endothelial cell lineage marker CD31 were excluded [36,37]. The GFP^+^ MSCs were combined with a wild-type bone marrow depleted MSC fraction and transplanted into syngeneic or allogeneic recipients. Furthermore, two syngeneic variants were used. One with self-bred transgenic mice and one with a commercially available strain.

### 2.1. Comparison between the Classic and New Methods of Purifying MSC Isolation

**Figure 1:**
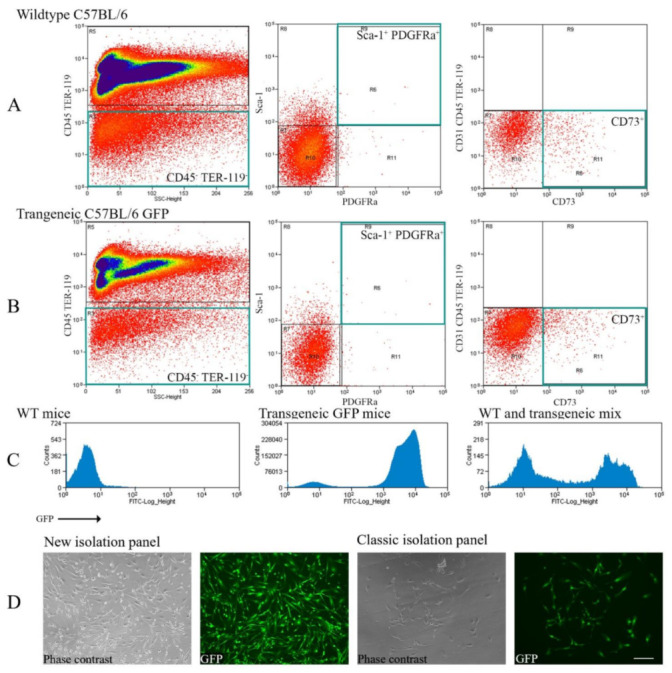
Comparison between the classic FACS staining panel and the new protocol to isolate freshly purified MSCs. (**A**) Displays whole bone marrow cells from wild-type C57BL/6 mice. (**B**) Shows whole bone marrow cells from transgenic GFP C57BL/6 mice. Green squares indicate the isolated populations. The staining panel were either CD45 TER-119 Sca-1^+^ PDGFR-α^+^ (**middle**) or CD31^−^ CD45 TER-119 CD73^+^ (**right**). (**C**) Exhibits the GFP signal in the wild-type (**left**), GFP transgenic (**middle**), and wild-type-transgenic mix (**right**, only for illustration). (**D**) Depicts isolated cells after 2 to 4 days of culture. The culture was only for illustrative purpose, not for transplantation. Scale bar = 100 µm, 200× magnification.

While the general preparation was identical, the isolation markers were slightly varied. This was mostly due to the weak signal of the PDGFR-α population (Figure 1, middle). Another issue was the close excitation wavelength of GFP and PE. Not every machine was able to remove the GFP signal from the PE channel, resulting in a PE signal in the unstained control of transgenic mice.

Hence, we tried a new set of antibodies to isolate MSCs in a more reliable and apparent way. The new set had the advantage of removing PE which had a partial overlap with GFP, as well as a stronger APC signal from CD73 compared to PDGFR-α (Figure 1A,B, middle vs. right). This way, between 25 and 50% more MSCs were isolated compared to PDGFR-α.

Several trials with the classic and new panel were undertaken and compared to validate the replicability and correctness of the isolation (Figure 1A–C). In addition, the cell morphology was confirmed by culturing MSCs on glass slides (Figure 1D).

### 2.2. Histochemistry: Detecting MSCs

After extracting the lacrimal gland from the corresponding mice, the tissue was fixated in 10% buffered formalin and embedded in paraffin. The sections were then subjected to Mallory staining or Haematoxylin & Eosin (HE) staining.

**Figure 2:**
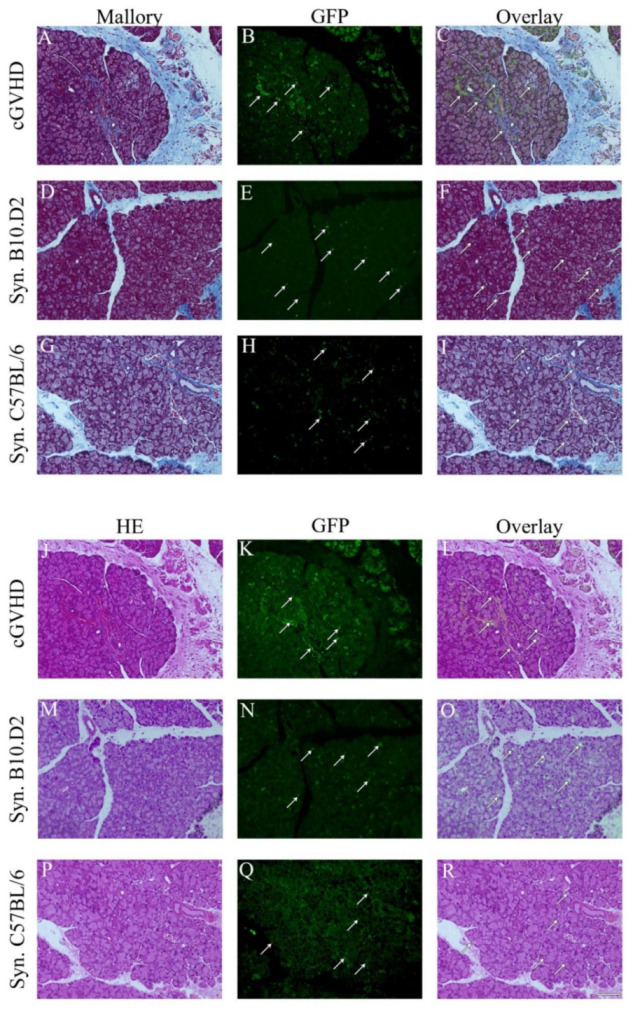
Mesenchymal stem cells become different phenotype after syngeneic and allogeneic MSC transplantation. Mallory (top **A**–**I**) and HE (bottom **J**–**R**) staining of the lacrimal gland in the allogeneic (cGVHD *n* = 19) and two syngeneic models (B10.D2 (*n* = 3) and C57BL/6 (*n* = 25) recipient mice). Scale bar = 100 µm, 200× magnification. Arrows indicate GFP^+^ cells. Mallory and HE staining are on the left side. The middle depicts the GFP channel and the right is a merge of both.

HE staining was used to confirm the GVHD phenotype. The typical inflammation and pathological changes were seen in various tissues (Supplementary Figure S1). Mallory staining in the allogeneic model revealed excessive fibrosis around the medium-sized ducts of the lacrimal gland typical of GVHD (Figure 2A). In contrast, fibrosis in both syngeneic models was comparatively low (Figure 2D). In the Mallory staining, GFP^+^ cells were clearly visible throughout the tissue (Figure 2B,E,H). GFP^+^ cells in the allogeneic model (cGVHD) appear to have integrated into the tissue while in syngeneic models the GFP^+^ cells retain a spherical shape. Similar findings were seen in the HE staining (bottom). There was no clear preferred site at which GFP^+^ cells resided or moved to.

In detail, many round-shaped cells were present in the syngeneic models (Figure 2E, H). In contrast, the allogeneic model displayed a massive presence of GFP cells. However, while round-shaped cells were also present, the majority contributed to structures and shapes of the tissue (Figure 2B). This phenomenon was especially found around blood vessels.

Yet, since the lacrimal gland is a secretary organ, the background signal can be high due to a variety of proteins in acinar epithelial cells. Consequently, an absolutely accurate assessment cannot be made from Mallory or HE staining. Adequate cells were defined as such if the signal’s shape was reasonable, of an appropriate size, and with a prominent signal.

### 2.3. Immunohistochemistry: Confirming the GFP Signal

**Figure 3:**
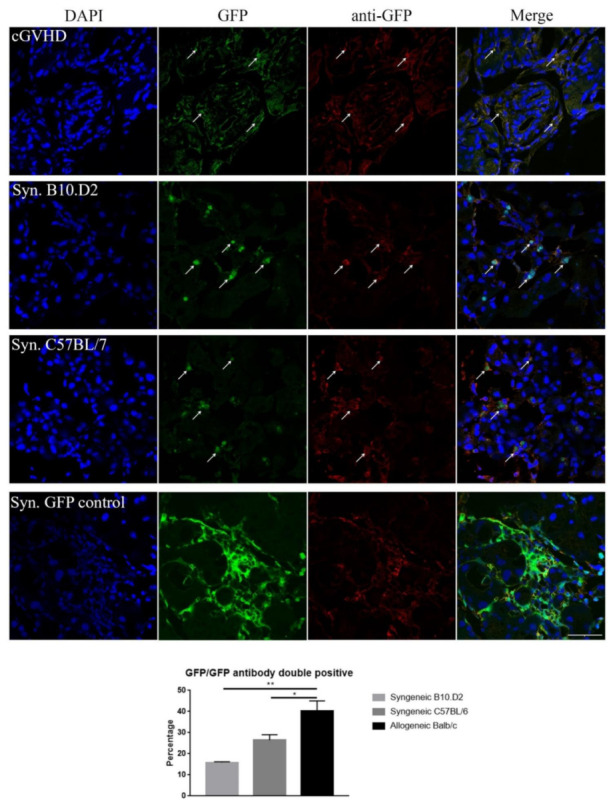
GFP control staining for the lacrimal gland tissue. Anti-GFP staining in allogeneic (top, *n* = 7), syngeneic (center, C57BL/6 *n* = 7, B10.D2 *n* = 3), and GFP^+^ whole bone marrow syngeneic (positive control, bottom) mice. Arrows indicate GFP signals. DAPI (blue), GFP (green), collagen type I (red), and double positive cells (yellow). Scale bar = 50 µm, 400× magnification. Bar diagram displays mean ± SEM, * *p* < 0.05, ** *p* < 0.01.

To further assess a reliable determination of the GFP^+^ cells, formalin-fixed frozen sections were prepared. Subsequently, the sections were stained with an anti-GFP antibody (Figure 3).

Cells that are emitting a GFP, as well as an anti-GFP antibody signal in close proximity to DAPI, stained nuclei were considered as being derived from transplanted GFP^+^ MSCs. Similarly, in the histochemistry, the syngeneic samples displayed a great number of spherical GFP^+^ cells that were distributed over the whole tissue (Figure 3, synC57BL/6, synB10.D2). The accuracy was confirmed with the GFP antibody. This result was consistent in both syngeneic models.

In contrast, when looking into the allogeneic model, spherical cells were rather rare. Instead, a general widespread distribution of GFP signals and spindle-shaped cells were observed across the tissue (Figure 3, cGVHD, GFP).

In comparison, the allogeneic model displayed approximately 60% more GFP^+^ cells compared to both syngeneic models, with 25 and 15 cells on average per picture. There was no significant difference detectable between the syngeneic C57BL/6 and B10.D2 model (Figure 3, Syn. rows, GFP).

Finally, to confirm these results, syngeneic recipient mice that received whole bone marrow transplantation from transgenic C57BL/6 GFP mice were used as a positive control (Figure 3, Syn. GFP control).

In addition, the lacrimal gland structure was highlighted by E-cadherin staining. No GFP signal was detected within the acinar epithelium (Supplementary Figure S2).

### 2.4. Immunohistochemistry: Investigating the GFP^+^ Cells

It seems evident that the transplanted GFP^+^ MSCs underwent a change in their phenotype in the lacrimal gland microenvironment. One of the hallmarks of GVHD is excessive fibrosis in target organs, including the lacrimal gland. MSCs on the other hand are known to have remodeling abilities [39,40], including collagen production [8,9,10,11]. Furthermore, Ogawa et al. [26] showed that freshly isolated MSCs are a major factor for GVHD progression.

**Figure 4:**
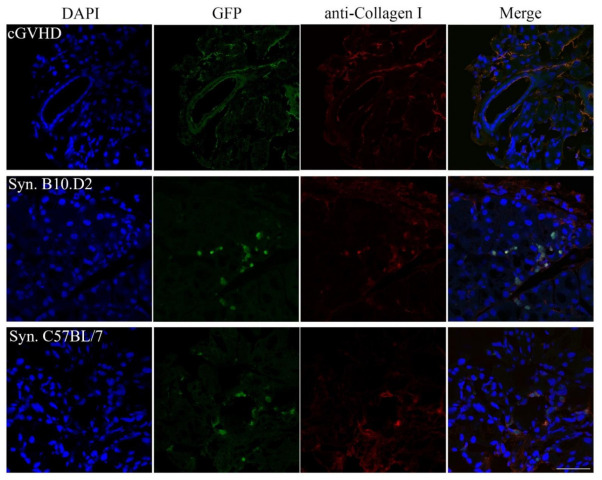
Anti-collagen type I staining in the lacrimal gland of GFP^+^ transplanted mice. Formalin-fixed frozen sections of recipient mice lacrimal glands were stained with an anti-collagen type I antibody. Representative figures from independent experiments (allogeneic *n* = 9, syngeneic C57BL/6 *n* = 9, B10.D2 *n* = 3). The allogeneic recipient (top), as well as both syngeneic models (middle and bottom), were collagen type I positive for most (>85%) of the GFP^+^ cells. DAPI (blue), GFP (green), collagen type I (red), and double-positive cells (yellow).Scale bar = 50 µm, 400× magnification.

Hence, there might be the possibility that the MSCs became activated excessive collagen-producing cells to drive pathogenic fibrosis in the lacrimal gland of GVHD. To test this hypothesis, formalin-fixed frozen sections were stained with an anti-collagen type I antibody. Anti-collagen type I staining revealed that 85% to 95% of the GFP cells in the allogeneic recipients were also collagen type I positive. This was also true for both syngeneic models (Figure 4).

### 2.5. Blood and BM Analysis

**Figure 5:**
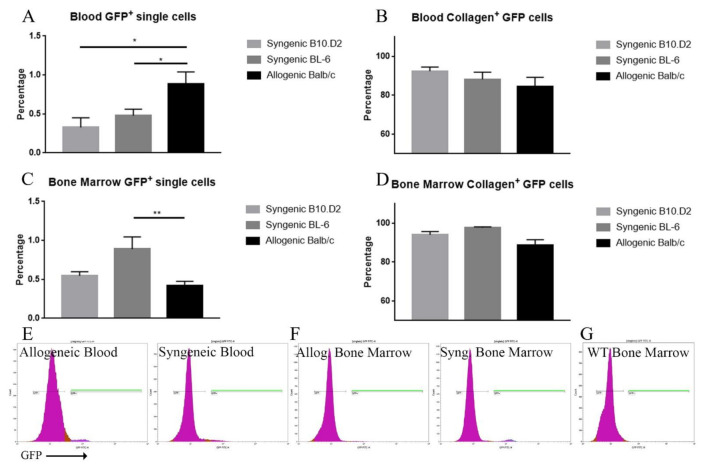
FACS analysis of the blood and bone marrow of the allogeneic and syngeneic models. The left side (**A**,**C**) depicts the percentage of GFP^+^ cells among the single sorted cells. The right side (**B**,**D**) depicts the percentage of how many GFP^+^ single cells are also collagen type I positive. While the GFP^+^ cells in the blood were comparatively higher in the allogenic model (**A**), the opposite was the case in the bone marrow (**C**). The majority of GFP^+^ cells were also positive for collagen type I (**B**,**D**). Data are presented as mean ± SEM, * *p* < 0.05, ** *p* < 0.001. Allogeneic *n* = 17, syngeneic BL/6 *n* = 17, syngeneic B10.D2 *n* = 10. (**E**,**F**) shows representative GFP signal figures. GFP^+^ gates are highlighted in green. (**G**) Displays a wild-type control as reference.

Finally, blood and bone marrow were obtained from the corresponding recipients on the acquisition day and analyzed by FACS. While there was no significant difference between the proportion of GFP^+^ cells which express collagen type I (Figure 5B,D), the overall amount of GFP^+^ cells was different among the three groups. In detail, when looking at the bone marrow, the allogeneic model had approximately 50% more GFP^+^ cells than syngeneic C57BL/6 (*p* = 0.007) and almost double compared to syngeneic B10.D2 (*p* = 0.343) (Figure 5C). In contrast, the blood showed the opposite effect (*p* = 0.035 and *p* = 0.066, respectively) (Figure 5A). Here, significantly more GFP^+^ cells were present in the allogeneic model, compared to both syngeneic models.

## 3. Discussion

The goal of this study was to investigate the role of donor-derived MSCs in murine whole bone marrow transplantation. For this purpose, a well-established cGVHD model was compared to two syngeneic models. The syngeneic models differed only in the strains used (C57BL/6 and B10.D2 namely). Transgenic B10.D2 mice were generated by us (see Section 4) and used as a same-strain syngeneic model. C57BL/6 mice on the other hand are commercially available and hence serve as a commercial model. This study focused on the lacrimal gland, one of the most frequently affected organs in cGVHD.

Firstly, the MSCs were isolated with a classic and a new staining panel by flow cytometry. Isolation of PDGFRα has shown to be more difficult due to a weak signal. Furthermore, the isolation of PE can be problematic when using GFP transgenic cells due to possible channel spillover. When comparing the yield and observed phenotype between the two panels, no difference was observable (Figure 1). Hence, it seems reasonable to assume both isolation methods resulted in the same MSC population.

Subsequently, HE, Mallory, and IHC were used to investigate whether GFP^+^ MSCs engrafted in the lacrimal gland tissue caused pathological differences (Figure 2). The presence of GFP throughout the allogeneic group was unexpected (Figure 2B,K). One major concern is the high protein/mucus production in the lacrimal gland. Therefore, it might seem that a high level of autofluorescence is caused by the proteins. However, two arguments speak against this assumption. Firstly, it needs to be taken into consideration that one of the hallmarks of transplantation, especially allogeneic, is dry eye disease. This is due to pathogenic changes within the lacrimal gland, resulting in decreased tear fluids and mucus production [41]. Furthermore, when comparing the allogeneic GFP pictures of HE or Mallory to the syngeneic ones, neither of the two syngeneic models display a remotely similar signal (Figure 2B,K vs. E,H,N,Q). In addition, it aligns with previous studies suggesting that donor MSCs are a major contributor to GVHD pathogenesis [42].

When looking at the areas where these cells were present, there seems to be no common factor. The cells are distributed among the tissue, regardless of inflammation, fibrosis, etc.

Interestingly, GFP^+^ cells were present in both, syngeneic and allogeneic models. However, the phenotype and distribution of GFP^+^ cells were much different. While in the allogeneic model, donor cells appear to be more integrated into the recipient’s tissue (Figure 2B,K and Figure 3, cGVHD), the GFP^+^ cells in the syngeneic model are more spherical (Figure 2E,H,N,Q and Figure 3, syngeneic). It may be that they are mobile and migrate through the tissue. Several studies suggest that syngeneic MSCs take immunomodulation functions in a suppressive way by inducing regulatory T cells (T reg) and suppressing T cell function [43,44,45]. In line with this, Ogawa et al. showed that in fact the host’s T cells partially contribute and collaborate with other cells for fibrosis [26]. Hence, it may be that while allogeneic MSCs integrate into the tissue, syngeneic MSCs communicate with the host’s T regs and suppress pathogenic T cell function. Further analysis of immune cell response might shed more light on this matter.

Another interesting aspect is the detection of collagen type I in most (>85%) GFP^+^ cells (Figure 4). While the presence of collagen most likely does not modulate T reg recruitment, the time course must be taken into consideration. The aforementioned Treg study reported their results relatively soon after transplantation (seven days). Hence, it might be feasible that the MSCs differentiate after initially arriving at the tissue site to specify their properties. This behavior was seen by many studies before [46,47,48]. Furthermore, Waterman et al. demonstrated that toll-like receptor (TLR) 3 and 4 have a contrary effect on collagen expression in MSCs. On the other hand, both receptors promote migration. Hence, it would seem sensible to investigate the TLR-3 and TLR-4 expression and activation in MSCs [49].

Similarly, flow cytometry analysis revealed that in the blood, as well as the bone marrow the majority of GFP^+^ cells contained collagen type I in both models (Figure 5B,D). This might indicate that the majority of the donor’s MSCs pursue a similar purpose, however, the results are different. The allogeneic model on one hand has many GFP^+^ cells circulating through the body compared to syngeneic peripheral blood. At this point, it seems they are in a “roaming” state. On the other hand, the syngeneic model had more GFP^+^ cells residing in the bone marrow. However, taking the tissue sections into consideration, there are many GFP^+^ cells in the lacrimal gland as well in the allogeneic model. Hence, there must be more cells overall in the allogeneic model to be in the affected tissue as well as in the blood. There are two possibilities to explain this matter. Either the transplanted MSCs proliferate significantly stronger in the allogeneic model or they proliferate once they engraft at the target tissue. Concluding from the flow cytometry data, they most likely mobilize from the bone marrow into the inflammatory environment of the allogeneic model. Further analysis of this matter is mandatory.

In summary, these results suggest that the phenotype of allogeneic and syngeneic models of MSCs are different. While both appear to move into the lacrimal gland, the allogeneic MSCs engraft and become part of the tissue and spindle-shaped cells in the interstitial stroma. In contrast, many syngeneic MSCs display as spherical-shaped cells, suggesting they remain distinct from the surrounding tissue. While the allogeneic and syngeneic models show GFP-expressing cells in areas with collagen, the outcome seems different. The allogeneic model displays a pathogenic collagen production; the syngeneic model might rather maintain homeostasis. Furthermore, the substantially different morphology of the cells suggests that they are not the same types of cell anymore. It might be possible that the MSCs became different subtypes in both models due to the differences in the microenvironment upon arrival. Taken together, it may become more established that freshly isolated MSCs differ from cultured MSCs. Upon entering the lacrimal gland, the microenvironment affects the cells in a different way, depending on the model of transplantation. The spherical shape of the syngeneic transplanted MSCs suggests that these cells are mobile, either actively moving through or around the tissue. In contrast, the spindle-shaped cells in the allogeneic model suggest that the MSCs have integrated into the tissue. Either way, more investigation is needed. Since GVHD is a systemic disease, other tissues such as the lung, salivary gland, and spleen are primary locations. The lung is one of the initial tissues where the injected cells pass through, the salivary gland has similarities with the lacrimal gland, and finally, the spleen is a representative tissue of immune modulation. In this regard, other immune markers such as CD45 detect immune cells around the MSCs and Treg markers such as FoxP3.

Finally, stromal cells are generally involved in tissue homeostasis. MSCs, in particular, can influence the adaptive and innate immune response. However, the exact mechanisms and differences between MSCs, depending on tissue, origin, and pre-applicational treatment are still broadly unknown [49,50]. Many questions remain to be solved. Further investigations for the mechanistic insight will be essential for the future.

## 4. Materials and Methods

### 4.1. Mice

In this study, 8 to 10-week-old donor mice of the strains C57BL/6NCrSlc (syngeneic model), C57BL/6-Tg (CAG-EGFP) (syngeneic model), and B10.D2/nSnSlc (MHC H2-d) (syngeneic and allogeneic model; wild type and transgenic 10th generation) were used. Recipient mice were 8-week-old C57BL/6NCrSlc (syngeneic), B10.D2/nSnSlc (MHC H2-d) (syngeneic), and BALB/c CrSlc (MHC H2-d) (allogeneic) mice.

Hence, this is an MHC identical and minor antigen mismatched transplantation. Syngeneic transplantations of GFP transgenic B10.D2 mice (own breeding) into B10.D2, as well as GFP transgenic C57BL/6 (commercially available) into C57BL/6 mice, were used as control.

Procedures were handled in accordance with the Institutional Guidelines on Animal Experimentation at Keio University and the ARVO Statement for the Use of Animals in Ophthalmic Vision Research. The protocols were approved by the Keio University Institutional Animal Care and Use Committee (Ethic approval code: #09152, date: 13 February 2019).

### 4.2. MSC Isolation and Transplantation

MSCs were isolated as demonstrated by several groups before [36,37,38]. Briefly, the femur, tibia, and ilium of 8-week-old donor mice were isolated, washed by PBS (Nacalai Tesque, Inc., Kyoto, Japan), and crushed. The bone marrow cells were removed by a quick washing of the bone and cell suspension. Next, the bone fragments were further crushed and minced with scissors until it resembled a paste-like mass. This bone paste was incubated in 0.2% collagenase DMEM Ham’s F-12 (Nacalai Tesque) at 37 °C for 1 h with constant shaking.

For the syngeneic model, MSCs from C57BL/6-Tg (CAG-EGFP) mice (Japan SLC, Inc., Shizuoka, Japan) or B10.D2/nSnSlc mice (own breeding after 10th generation) were used. The latter was also used for the allogeneic model.

The MSC containing supernatant was collected and supplemented with additional washings of the minced bones. The washing and staining buffer was HBSS (Nacalai Tesque) with 1% penicillin/streptomycin (Nacalai Tesque), 2% Fetal Bovine Serum (JRH Biosciences, Lenexa, KS, USA), and 10 mM HEPES (Fujifilm Wako Pure Chemical Cooperation, Tokyo, Japan). Red blood cells were lysed with water (Nacalai Tesque). Subsequently, the cells were stained with either CD31-PECy7, CD45-PECy7, TER-119-PECy7, CD73-APC or CD45-PECy7, TER-119-PECy7, PDGFRα-APC, and Sca-1-PE (see Table 1). The first one is the new and the latter the classic composition.

Cells were isolated with the MoFlo XDP Cell Sorter (Beckman Coulter, Tokyo, Japan). Here, MSCs were defined as CD45^-^ TER-119^-^ Sca-1^+^ PDGFRα^+^ (a classic marker for MSCs) or CD31^-^CD45^-^TER^-^119^-^CD73^+^ (a new marker for MSC).

Furthermore, wild-type bone marrow was obtained from C57BL/6NCrSlc (Japan SLC, Inc., Japan) or B10.D2/nSnSlc (Japan SLC, Inc., Japan), respectively. The isolated whole bone marrow was deprived of MSCs by sorting for CD31^+^CD45^+^TER-119^+^ cells.

The gating was verified with fluorescence minus one and isotypes.

A mixture of 2 × 10^4^ GFP^+^ MSCs and 1 × 10^6^ MSC depleted whole bone marrow was injected into 7.5 Gy irradiated mice. The recipients were C57BL/6NCrSlc and B10.D2/nSnSlc (Japan SLC, Inc., Japan) for syngeneic transplantation, respectively. For the allogeneic model, BALB/cCrSlc (Japan SLC, Inc., Japan) were used.

As a positive control, irradiated C57BL/6NCrSlc mice were injected with whole bone marrow from C57BL/6-Tg (CAG-EGFP) GFP mice.

### 4.3. MSC Culture

After isolation, cells were cultured on a standard six-well plate or on holed microscope glass slides (Matsunami Glass Cooperation, Osaka, Japan) and Dulbecco’s Modified Eagle Medium (DMEM) with 20% fetal bovine serum (FBS), 16 ng/mL fibroblast growth factor, and 1% P/S.

### 4.4. Histochemistry

Histochemistry was performed with 10% buffered formalin-fixed, paraffin-embedded samples. The fixation time was 24 h. Samples were thoroughly deparaffinized with xylene and gradually rehydrated with decreasing concentrations of ethanol with the last step being pure distilled water.

Hematoxylin and Eosin (HE) staining (Nacalai Tesque) was performed as follows: deparaffinized samples were immersed in hematoxylin stain until they reached the desired intensity. Every 10 s the staining was assessed through the microscope. After washing, the slides were immersed in 0.1% eosin stain for 5 min. After a brief prewashing with 50% ethanol, the samples were quickly dehydrated with an increasing ethanol concentration. Thereafter, xylene was used as the final step before mounting.

Mallory staining was performed as recommended by the manufacturer’s protocol (Sigma-Aldrich, Tokyo, Japan), except the incubation time for the Biebrich Scarlet-acid fuchsin and aniline blue solution was increased to 10 min. After staining the samples were dehydrated as described above and mounted. All pictures (white light and GFP) were taken with Biorevo BZ-9000 (Keyence, Tokyo, Japan).

### 4.5. Immunohistochemistry

The tissues were fixed with 10% buffered formalin for 3 h, then washed after fixation briefly, and transferred to OCT compound (Sakura Finetek Japan, Co., Ltd., Tokyo, Japan) and frozen at −80 °C. The frozen samples were cut with a cryostat and covered with Normal Goat Serum (Thermo Fisher, Waltham, MA, USA) or 10% Normal Donkey Serum (Merck, Kenilworth, NJ, USA) in PBS for 45 min at room temperature to minimize background signaling.

Alternatively, paraffin sections were deparaffinized and treated with antigen retrieval (Dako, Carpinteria, CA, USA). Subsequently, the samples were stained with an anti-collagen I or anti-GFP antibody (see Table 1) overnight at 4 °C. The following day, the secondary antibody Alexa Fluor 555 was added for 45 min at room temperature. After washing, the cells were mounted with DAPI and anti-fading reagent containing mounting medium. All pictures were taken with a Leica LSM 710 microscope (Leica, Wetzlar, Germany).

### 4.6. Flow Cytometric Analysis for Collagen Type I Production by MSCs

Blood and bone marrow were taken on the acquisition day. The red blood cells were lysed with water (Nacalai Tesque) before staining. Bone marrow cells were cultured overnight in 6 cm FNC (Nacalai Tesque)-coated plastic dishes in DMEM. The following day, the supernatant was removed and only the attached cells were stained.

The staining procedure was done in three steps: first, the cells were fixated with 1%PFA in PBS (Nacalai Teque) for 10 min at room temperature. The cells were washed several times with a 1% saponin-containing flow cytometry buffer. Next, the cells were stained with anti-collagen type I antibody (see Table 1). After 1 h of incubation time on ice, the cells were washed with saponin-containing buffer and stained with anti-rabbit PE secondary antibody (see Table 1).

The gating was verified with wild-type cells for GFP and anti-rabbit PE without collagen antibody.

### 4.7. Statistics

Statistical significance was assessed with unpaired, two-tailed t-tests. A *p*-value of *p* < 0.05 was considered as significant.

## Figures and Tables

**Table 1:** Antibody list.

Name	Clone	Reporter	Species	Company
CD 31	MEC13.3	PE-Cy7	Rat	Biolegend
CD 45	30-F11	PE-Cy7	Rat	Biolegend
TER-119	TER-119	PE-Cy7	Rat	Biolegend
PDGFRa	APA5	APC	Rat	Biolegend
Sca-1	D7	PE	Rat	Biolegend
CD 73	17A2	APC	Rat	Biolegend
E-Cadherin	24E10	none	Rabbit	Cell Signaling
anti-GFPuv	polyclonal	none	Goat	R&D Systems
Anti-Collagen Type I	polyclonal	none	Rabbit	Abcam
Donkey anti-Rabbit IgG	Polyclonal	Alexa Fluor^®^ 555	Donkey	Invitrogen
Donkey anti-Goat IgG	Polyclonal	Alexa Fluor^®^ 555	Donkey	Invitrogen
Donkey anti-Rabbit IgG	Polyclonal	PE	Donkey	Biolegend
PE isotype	RTK2758	PE	Rat	Biolegend
PE-Cy7 isotype	RK4530	PE-Cy7	Rat	Biolegend
APC isotype	RTK2758	APC	Rat	Biolegend

## Data Availability

The data that support the findings of this study are available within the article and supplemental data or from the corresponding author upon reasonable request.

## Supplementary Materials

Supplementary materials can be found at https://www.mdpi.com/article/10.3390/ijms22094895/s1.
