# Supplementary material for: MSCs Become Collagen-Type I Producing Cells with Different Phenotype in Allogeneic and Syngeneic Bone Marrow Transplantation
Source: Int J Mol Sci. 2021 May 5;22(9):4895. doi: 10.3390/ijms22094895 (PMC8125797; doi:10.3390/ijms22094895)
Supplement: Supplementary file 1 [file ijms-22-04895-s001.zip › ijms-1199972-supplementary.pdf]

Supplementary Materials

MSCs Become Collagen-Type I Producing Cells with Different Phenotype in Allogeneic and Syngeneic Bone Marrow Transplantation

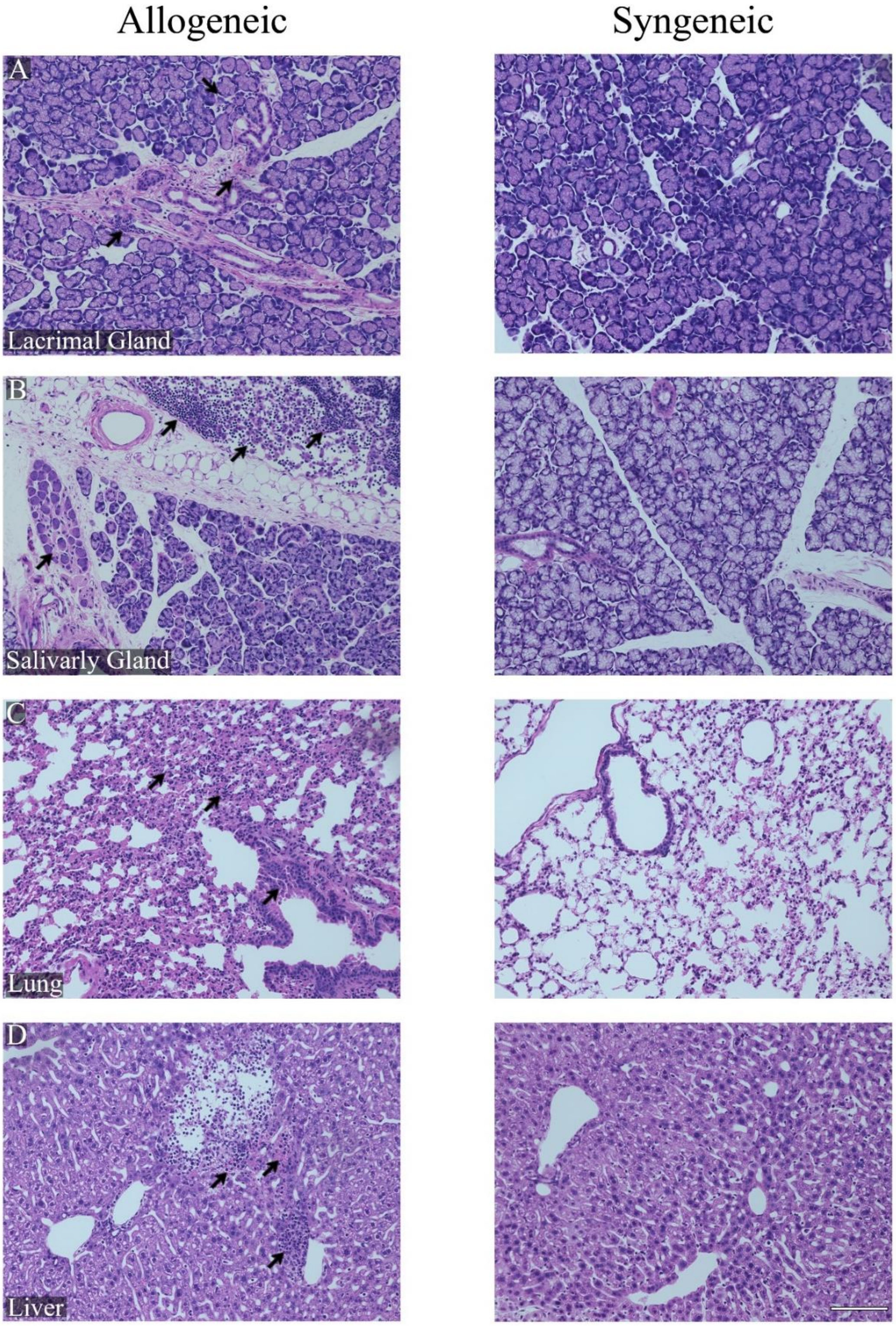

Figure S1. Inflammatory cell infiltration in graft-versus-host disease (GVHD) mouse model. Hematoxylin & Eosin (HE) staining of the lacrimal gland (A) shows early signs of tissue infiltration of inflammatory cells as a typical early sign of GVHD. In the salivary gland (B) some lobuli are destroyed by excessive inflammatory cell infiltration, or show initial signs of acinar damage. The lung (C) displays a reduced alveolar space due to excessive inflammatory cell infiltration and fibrosis. In the liver (D) inflammatory cells are observed around bile ducts.

Some areas are destroyed due to severe inflammation. Taken together those findings suggest the GVHD phenotype is clearly observed in the target organs. 200x magnification, scale bar = 100μm.

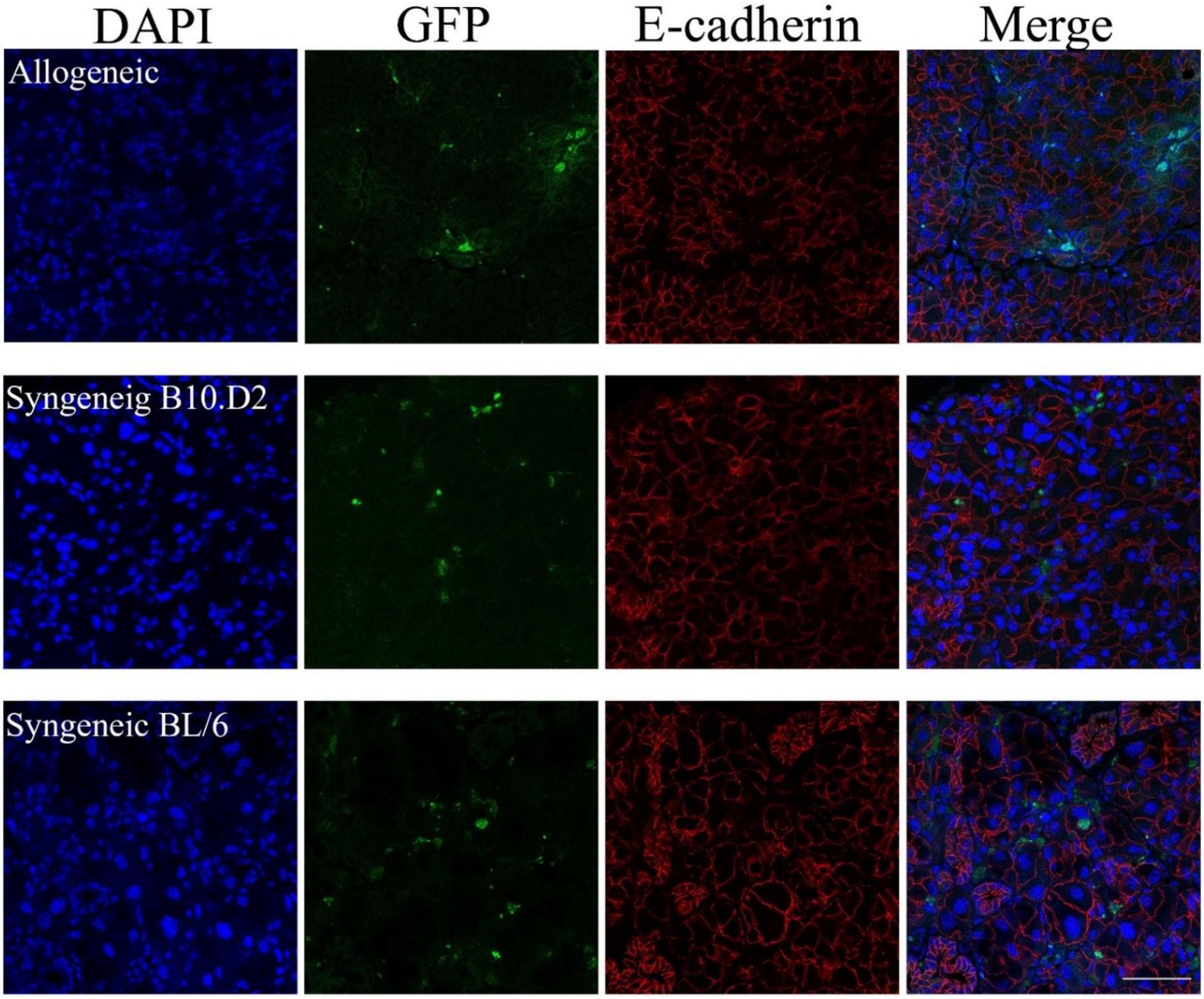

Figure S2. GFP[+] donor-derived cell engraftment in lacrimal gland of allogeneic or syngeneic mesenchymal stem cell transplanted recipient. Samples were stained with DAPI (blue) and E-cadherin (red) to highlight the structure of the lacrimal gland. GFP cells (green) were detected in all samples. GFP[+] cells were not detected within the acinar epithelium. 400x magnification, scale bar = 50μm.
